# Occupational Therapy for Parkinsonian Patients: A Retrospective Study

**DOI:** 10.1155/2019/4561830

**Published:** 2019-11-03

**Authors:** Michele Franciotta, Roberto Maestri, Paola Ortelli, Davide Ferrazzoli, Federica Mastalli, Giuseppe Frazzitta

**Affiliations:** ^1^Department of Parkinson's Disease, Movement Disorders and Brain Injury Rehabilitation, Moriggia Pelascini” Hospital, Gravedona ed Uniti, Italy; ^2^Istituti Clinici Scientifici Maugeri IRCCS, Department of Biomedical Engineering of the Montescano Institute, Italy; ^3^MIRT ParkProject, Livorno, Italy

## Abstract

**Background:**

Hand functionality and finger dexterity are impaired in patients with Parkinson's disease (PD). These disturbances lead to a dependency in activities of daily living (ADL) and poor quality of life (QoL).

**Objective:**

We aimed to evaluate whether a specific occupational therapy (OT) program is effective in improving finger and hand dexterity and its impact on ADL in PD patients.

**Methods:**

We retrospectively studied PD patients, hospitalized for a 4-week multidisciplinary intensive rehabilitation treatment (MIRT) between January 2015 and June 2018. All patients underwent 1 h/day OT treatment, 5 days a week. The primary outcome measure was the O'Connor finger dexterity test; secondary outcome measures were the Minnesota dexterity test, UPDRS II, and Self-Assessment Parkinson's Disease Disability Scale (SPDDS). These measures were assessed at admission (T0) and discharge (T1).

**Results:**

Based on the Hoehn and Yahr scale (H&Y), patients were divided into two groups: 262 subjects in H&Y stage <3 (early-stage PD patients) and 220 in H&Y stage ≥3 (medium-advanced stage PD patients). As expected, at baseline, all measures were worse in higher H&Y stages. After treatment, both groups experienced significant improvements in all outcomes. Significant differences between early-stage and medium-advanced stage PD patients were observed only for the changes in UPDRS II, with a better improvement in patients in H&Y stage ≥3.

**Conclusions:**

We showed that PD patients who underwent a rehabilitation protocol including OT experienced improvements in finger dexterity and hand functionality. Our results underline the relevance of OT in improving autonomy and QoL in PD patients.

## 1. Introduction

Hand dysfunction is a common symptom in Parkinson's disease (PD) and is characterized by (i) poor manual dexterity, (ii) deficits in fine motor movements, (iii) inability to control grip force output, and (iv) difficulty in performing movements with normal amplitude, speed, and coordination [1, 2].

Hand dysfunction leads to difficulties in activities of daily living (ADL), such as eating, dressing, washing, and writing [3], with loss of independency and poor quality of life (QoL).

These abilities are acquired through motor learning processes that are related to executive functions [4, 5].

Despite the presence of deficits in hands functionality and the reduced autonomy in ADL [6], these disturbances are often ignored because the majority of rehabilitation programs are focused on gait and balance problems [7, 8]. Therefore, patients are usually referred to an occupational therapist in the later disease stages, when they are experiencing a significant level of disability [9–11].

Only few studies have evaluated the effectiveness of occupational therapy (OT) for patients with PD [12]. Several multidisciplinary rehabilitative treatments designed for Parkinsonian patients have been shown to be effective in reducing PD symptoms and improving motor functions [8, 13–15].

OT aims at treating hand impairment to reduce dependency or recover the patients' autonomy in ADL [6, 9], and it should be considered as an important aspect of a multidisciplinary approach.

No previous studies have investigated the effectiveness of an intensive, hand-based OT treatment, designed as a part of a multidisciplinary-integrated approach, on hand functionality and autonomy in daily living in patients with PD.

We aimed to evaluate whether (i) a specific OT is effective to improve finger and hand dexterity in PD patients, (ii) the effects of OT differ among patients at different disease stages, and (iii) a better hand function leads to improvements in personal autonomy.

## 2. Materials and Methods

### 2.1. Study Design and Participants

We retrospectively identified PD patients, hospitalized at the Department of Parkinson's disease and Movement Disorder Rehabilitation of “Moriggia-Pelascini” Hospital in Gravedona ed Uniti (Como, Italy), from January 2015 to June 2018, to undergo a 4-week Multidisciplinary Intensive Rehabilitation Treatment (MIRT) [14, 15].

  Eligibility criteria were as follows: (1) diagnosis of idiopathic PD according to the UK Brain Bank criteria [16]; (2) Hoehn and Yahr (H&Y) stage 2 to 4; (3) ability to perform both the O'Connor finger dexterity test [17] and the Minnesota manual dexterity test [18].
  Exclusion criteria were as follows: (1) diagnosis of atypical parkinsonisms, (2) psychosis, (3) auditory and visual dysfunctions, and (4) comorbidities impairing autonomy in ADL.

The study design and protocol were approved by the local Ethics Committee (“Comitato Etico Interaziendale delle Province di Lecco, Como e Sondrio”) and were in accordance with the code of Ethics of the World Medical Association (Declaration of Helsinki, 1967). All patients signed an informed written consent form for the use of their clinical data for scientific purposes. This trial was registered on ClinicalTrials.gov website NCT03763955.

### 2.2. Outcome Measures

#### 2.2.1. Primary Outcome Measure

*(1) O'Connor Finger Dexterity Test*. The O'Connor finger dexterity test (O'CT) is a peg-placement test that has been used to evaluate the rapid manipulation of small objects. It consists of a Masonite board with a moulded surface consisting of 100 holes (each measuring 0.47 cm in diameter) arranged in ten rows, each containing ten holes. Holes are spaced 1.26 cm apart. Three-hundred and fifteen pins (length 2.54 cm; diameter 0.16 cm) lay in a well. The subject is required to fill each hole with 3 pins as fast as possible using his/her dominant hand. Time elapsed for filling the first half of the board (5 rows and 50 holes) is recorded in seconds and then summed to the time elapsed for filling the second half of the board (5 rows and 50 holes). The number of seconds taken to fill the second half of the board is multiplied by 1.1. The mean of this value and the number of seconds taken to fill the first half of the board are computed, i.e., raw score = (time for first 50 holes + (time for second half holes *x* 1,1))/2.

The test is considered as invaluable when patients need more than 15 minutes to fill the first five rows. The lower the score obtained by the patient, the better his/her clinical conditions. The O'Connor test was administrated by an occupational therapist at T0 (admission) and T1 (discharge, 4 weeks after T0).

#### 2.2.2. Secondary Outcome Measures

*(1) Minnesota Manual Dexterity Test*. The Minnesota manual dexterity test (MMDT) investigates reach-to-grasp movements, gross manual dexterity, and left vs right hand impairment through a both-hand handling task. The MMDT includes two test batteries: placing test and turning test. *Placing test* is performed with the dominant hand: it consists of placing 60 black and red plastic disks into a grid of four rows and aims at evaluating the upper limb speed in reach-to-grasp movements and the gross hand ability to manipulate object. In the *turning test*, the subject is asked to pick up, turn upside down, and put down each disk in the grid: these actions have to be performed by using one hand to pick it up and the other one to turn it upside down and put it back into the grid; the first part of the test (first two rows) is performed using the same turning hand, while in the second part (last two rows), subjects are requested to switch the hands' roles. The aim of the turning test is to evaluate the coordination skills and to highlight the difference between the affected limb and the unaffected one, by testing them during a both-hand handling task; the score is expressed in seconds. The lower the score obtained by the patient, the better his clinical conditions.

MMDT was administrated by an occupational therapist at T0 (admission) and T1 (discharge, 4-week after T0).

*(2) SPDDS (Self-Assessment Parkinson's Disease Disability Scale)* [19]. The SPDDS is a self-assessment, patient-centred questionnaire, aimed at measuring the disability in daily life of patients with PD. The questionnaire score ranges from 25 to 125 points. The lower the score, the better the clinical conditions. SPDDS was collected at T0 (admission) and T1 (discharge, 4-weeks after T0).

*(3) UPDRS (Unified Parkinson's Disease Rating Scale)* [20]. The UPDRS is the most used scale to assess the clinical severity of PD. It consists of four sections: I: mentation, behaviour, and mood (4 items); II: ADL (13 items); III: motor examination (14 items); and IV: complications of therapy (11 items). The UPDRS was applied by a neurologist with experience in movement disorders at T0 (admission) and T1 (discharge, 4-weeks after T0). The lower the score, the better the clinical conditions.

### 2.3. Neuropsychological Assessment

At admission, a neuropsychologist assessed the patients' cognitive profile using the frontal assessment battery (FAB), a test designed for evaluating executive functions (normal > 14 points).

### 2.4. QoL Assessment

At admission, patients were required to fill in the self-administered Parkinson disease's Questionnaire 39 (PDQ39), a questionnaire specifically designed to assess quality of life [21]. The lower the score, the better the clinical conditions.

### 2.5. Interventions

#### 2.5.1. MIRT Protocol

MIRT is a multidisciplinary, integrated, aerobic, motor-cognitive, intensive, and goal-based rehabilitation treatment specifically designed for patients with PD [13–15]. The aim of the treatment is to relearn the dysfunctional movements resulting from the disease through the use of explicit and implicit learning strategies. It consists of a 4-week program in a hospital setting, composed of four different rehabilitative sessions from Monday to Friday and 1 hour of physical exercise on Saturday. On Sunday, the patients have a rest day. The duration of each session, including recovery periods, is about 1 hour:

1. The first session consists of a one-to-one treatment with a physical therapist. It includes cardiovascular warm-up activities, active and passive exercises to improve joints' range of motion, stretching of the abdominal muscles, strengthening of paravertebral muscles, postural changes, and exercises operating on balance and postural control.
2. During the second session, patients are trained with different devices to improve gait and balance: a stabilometric platform with a biofeedback [22], a treadmill plus [23] (treadmill training with visual cues, auditory cues and feedback), a crossover [22], and a cycloergometer with feedback. We use a maximum treadmill speed of 3.5 km/hour; patients are trained with treadmill two times per day and each session lasts no more than 15 minutes.
3. The third is an OT session (see section 2.5.2).
4. The fourth session includes 1 hour of speech therapy [24].

The rehabilitation program might include hydrotherapy, robotic-assisted walking training, virtual reality training, and psychoeducational groups. During the activities, the heart rate reserve is kept between 70% and 80%.

Weekly team meetings are scheduled to tune the rehabilitation programme for each patient and to assess its benefits during the course of hospitalisation.

#### 2.5.2. Occupational Therapy Session

The OT session consists of a group session (1 h/day with no more than 5 patients per session) conducted and monitored by an occupational therapist, which tailors the rehabilitative project taking into account patients' individual motor and cognitive residual abilities. OT is centred on the difficulties encountered by the subject in everyday life. During the training sessions, patients undergo functional and goal-based exercises aimed at readapting the use of daily tools on the basis of their residual abilities and relearning how to perform everyday tasks for increasing personal autonomy. To enhance the hand functionality and the motor control of hand movements, every OT session is focused on finger and hand dexterity and coordination and dual tasking skills. The exercises are mainly designed to intervene on the side (hand) most affected by the disease, as well as on strategies for enhancing both-hands handling coordination. For this reason, a useful method adopted to intervene on the hand movements and functionality is the writing-skill training: it consists of pencil-and-paper exercises in which visual cues and verbal strategies are used to achieve an enlargement of letter size and improve the text readability. The rehabilitation protocol exploits motor, cognitive, and behavioural strategies and allows training of patients' awareness about their self-management potentialities in everyday life. For this reason, patients are asked to learn and perform simple exercises feasible also at home. Lastly, the occupational therapist explains the caregivers how to interact with the patient in the daily living to promote the subject's autonomy and increase their safety level.

### 2.6. Statistical Analysis

Descriptive statistics are reported as mean ± SD for continuous data and as number and percentage frequency for discrete variables. The Shapiro–Wilk test, supported by visual inspection, was used to assess the normality of the distribution of continuous variables. Since most outcome measures severely violated the normality assumption, nonparametric statistics were used. Accordingly, between- and within-group comparisons for continuous variables were performed by the Mann–Whitney *U* test and Wilcoxon signed-rank test, respectively. Comparisons of categorical variables were carried out with the chi-squared test or Fisher's exact test when appropriate.

We first compared the outcome variables at discharge with values at admission to assess the effect of MIRT on the overall population. The association between improvement in outcome measures (difference between values at discharge and values at admission) and demographic and clinical variables was investigated by Spearman's rank correlation coefficient. To further investigate whether MIRT affects positively the rehabilitation outcome and determine the benefit regardless of the patients' stage of disease (early-medium or advanced), we grouped patients according to H&Y < 3 or H&Y ≥ 3 and compared between-group improvement.

All statistical tests were two-tailed, and statistical significance was set at *p* < 0.05. When appropriate, false discovery rate was controlled at 5% using the Benjamini–Hochberg method. All analyses were carried out using the SAS/STAT statistical package, release 9.2 (SAS Institute Inc., Cary, NC, U.S.A.).

## 3. Results

The study population consisted of 482 patients; among them, 262 were in H&Y stage <3 and 220 were in H&Y stage ≥3. Demographical, neuropsychological data and amount of l-dopa equivalent dosage (LED) for the overall population and for both groups of patients are reported in Table 1. As expected, patients' mean age, LED, disease duration, and PDQ39 increased (i.e., worsened) passing from lower to higher H&Y stages, while years of education and FAB decreased (i.e., worsened).

In Table 2, the values of the clinical, motor, and functional outcome variables at baseline for all patients as a whole and grouped in H&Y < 3 and in H&Y stage ≥3 are reported, with *p* values for between-group comparisons. As expected, all outcomes at baseline significantly worsened passing from lower to higher H&Y stage (*p* < 0.0001 all, after Benjamini–Hochberg adjustment).

Table 3 reports the differences (values at discharge—values at admission) of the outcome measures. After MIRT, all outcome measures improved (i.e., decreased, for all these measures, the lower the better) significantly (i.e., the difference was significantly different from 0) in the overall population and in both groups of patients (adjusted *p* < 0.0001 all).

Comparing the amount of improvement in the two groups, significant differences were observed only for the changes in UPDRS II (adjusted *p* < 0.0001), with a better improvement in more advanced H&Y stages (−4.9 ± 2.5 vs −3.9 ± 2.1).

Correlation analysis revealed that the improvement in outcome measures assessing dexterity was not associated with H&Y stage, age, FAB, and PDQ39 (all adjusted *p* values >0.1). The improvement in UPDRS II was associated with H&Y stage, PDQ39 (adjusted *p* values <0.001 both), and with FAB (adjusted *p* values = 0.02), while no significant association was observed between SPDDS and H&Y stage, age, FAB, and PDQ39.

Finally, subdividing all patients based on the side of motor symptoms predominance (Table 4), we found differences between groups at T0 (nor at T1), only in placing test part 2 and total score (*p*=0.042 and *p*=0.036, respectively).

## 4. Discussion

This is the first study to evaluate the efficacy of a specific OT intervention, designed as part of a multidisciplinary, intensive, and integrated rehabilitation treatment, on hand functionality, and autonomy in ADL in people with PD. Our results indicate that both patients in early and in medium-advanced H&Y stage obtained benefits from OT, as they improved in fine hand movements, coordination, and autonomy in daily living.

Benefits in finger dexterity (evaluated with O'CT), as well as in hand dexterity and upper limb motor function (evaluated with MMDT), were observed. In a previous study, Taghizadeh and colleagues [25] investigated the effects of a sensory-motor training (2 week-treatment, 2 hours/day for 5 days/week) on hand and upper extremities in 50 PD patients (H&Y stage 1–3), showing that the treatment was effective. Lee and colleagues [26] reported improvements in fine and gross motor performances of the upper limb in PD patients (H&Y stage 2-3) after a 4-week period (3 hours/day for 5 days/week) of constraint-induced movement therapy. Mateos-Toset and colleagues in 2015 [27] demonstrated in 60 PD patients (H&Y stage 2-3) an improvement in manual dexterity and strength also after a single 15-minute hand-exercise session. Our results confirm that a goal-based, motor-cognitive training leads to improvements in hand and finger dexterity in PD patients [28].

We also found that both patients with right- and left-side motor symptoms predominance showed similar performances at the beginning of the treatment and gained similar benefit at discharge. Since 97% of our sample was composed of right-handed persons and the primary outcome measure (O'CT) was determined using the dominant hand, our data suggest that finger and hand dexterity are impaired in the dominant hand, regardless of the side of motor symptoms predominance. This finding is in line with previous data, showing that dopamine deficit in the right caudate correlates with difficulty in bimanual hand movements [29].

Despite a Cochrane review concluded that the evidence supporting the efficacy of OT in PD is insufficient [30], previous studies supported this approach as effective in reducing the functional impact of PD in daily life [9, 10]. Coherently, the improvements in UPDRS II and SPDDS scores suggest that MIRT is effective in improving the autonomy in daily living.

Our results differ from those obtained in other studies assessing the effects of a low-intensity program of physiotherapy and OT [11], thus underlining the importance of the treatment-intensity and specificity to obtain significant results in PD rehabilitation [31].

Strong correlations among hand dexterity, functional gains, and improvements in both SPDDS and UPDRS II were found. This is an important perspective in terms of self-perceived QoL that has been already been shown to improve after MIRT [32].

Our data confirm the efficacy of a multidisciplinary rehabilitation treatment on patients with PD [13–15] and highlight the contribution of hand training and OT.

In line with previous data [28, 33], we found a negative correlation between all outcomes and cognitive status as assessed by the FAB and a lack of association between cognitive status and the size of the improvement after rehabilitation. This is a noteworthy aspect: although patients with worse cognition are characterized by poorer motor performances, the cognitive status does not hamper the patients to achieve benefits after rehabilitation.

As expected, clinical conditions of patients in H&Y stage ≥3 were worse than those in H&Y stage <3. However, at discharge, patients in H&Y stage ≥3 showed a greater improvement in comparison with those in H&Y stage <3, likely due to a “ceiling” effect. These data confirm that, even though patients' performances are influenced by their motor and cognitive conditions, these aspects do not have a negative impact on rehabilitation outcomes [28, 33].

In conclusion, we showed that a specific OT intervention, designed as part of a multidisciplinary, intensive, and integrated rehabilitation treatment, is effective to improve finger and hand dexterity and autonomy in activities of daily living in PD patients, regardless of the disease severity. Our results underline (i) the relevance of OT, often neglected in the rehabilitative protocols for PD, and (ii) the need to start this treatment as soon as possible to prevent or delay disturbances of hand functioning.

This study has some limitations that have to be acknowledged: (i) the lack of a control group of patients undergoing a conventional, nonintensive, nor goal-based treatment and not including a specific OT program; (ii) we did not collect follow-up data to evaluate whether and how long the improvements observed in hand functionality and ADL last. Further studies are needed to clarify these issues and better clarify the role of OT in PD rehabilitation.

## Figures and Tables

**Table 1:** Patients' demographical, clinical, and neuropsychological data.

Variable	All	H&Y < 3	H&Y ≥ 3	*p*
Age (years)	67.1 ± 9.4	64.3 ± 10.2	70.3 ± 7.3	<0.0001
Male gender (%)	54.4	50.9	57.1	0.17
Right side (%)	55.3	47.8	61.6	0.004
Right-handed (%)	97.1	98.6	95.8	0.065
LED (mg)	618 ± 319	538 ± 294	711 ± 324	<0.0001
Disease duration (years)	8.9 ± 5.2	7.7 ± 5.0	10.2 ± 5.2	0.0002
H&Y	2.6 ± 0.5	2.2 ± 0.3	3.1 ± 0.3	<0.0001
Years of education	10.5 ± 4.4	11.0 ± 4.2	9.9 ± 4.6	0.002
FAB	13.9 ± 3.0	14.4 ± 2.8	13.2 ± 3.1	<0.0001
PDQ39	41.1 ± 22.1	34.8 ± 17.4	51.6 ± 24.9	<0.0001

LED: daily levodopa equivalent dose; H&Y: Hoehn and Yahr stage; FAB: frontal assessment battery; PDQ39: Parkinson disease's Questionnaire 39.

**Table 2:** Baseline values of the clinical, motor, and functional outcome variables for all patients as a whole and grouped in H&Y < 3 and in H&Y stage ≥3, with *p* values for between-group comparisons.

Variable	All patients T0	H&Y < 3	H&Y ≥ 3	*p*
O'CT (s)	499.6 ± 133.4	466.9 ± 117.6	541.5 ± 141.1	<0.0001
MMDT placing test 1 (s)	103.5 ± 23.4	97.9 ± 18.7	110.3 ± 26.5	<0.0001
MMDT placing test 2 (s)	97.8 ± 23.3	92.8 ± 19.5	103.8 ± 26.0	<0.0001
MMDT placing test total score (s)	201.2 ± 45.9	190.3 ± 37.9	214.3 ± 51.1	<0.0001
MMDT turning test 1 (s)	24.2 ± 8.6	22.1 ± 6.3	26.7 ± 10.3	<0.0001
MMDT turning test 2 (s)	25.6 ± 9.5	23.2 ± 6.7	28.5 ± 11.5	<0.0001
MMDT turning test 3 (s)	21.8 ± 7.0	20.0 ± 5.6	24.1 ± 7.9	<0.0001
MMDT turning test 4 (s)	22.9 ± 7.8	20.9 ± 6.1	25.2 ± 9.1	<0.0001
MMDT turning test total score (s)	94.2 ± 30.2	86.0 ± 22.4	104.2 ± 35.3	<0.0001
UPDRS part II	14.3 ± 5.0	12.1 ± 4.0	16.8 ± 4.8	<0.0001
UPDRS part III	19.8 ± 5.5	17.9 ± 4.5	21.9 ± 5.8	<0.0001
SPDDS	72.0 ± 13.6	68.8 ± 12.6	75.8 ± 13.9	<0.0001

O'CT (O'Connor finger dexterity test); MMDT (Minnesota manual dexterity test); UPDRS (unified Parkinson's disease rating scale); SPDDS (self-assessment Parkinson's disease disability Scale). Reported *p* values are from the Mann–Whitney *U* test (between-group comparison, H&Y ≥ 3 vs H&Y < 3).

**Table 3:** Differences (values at discharge—values at admission) of the outcome measures.

Variable	All patients	H&Y < 3 T1-T0	H&Y ≥ 3 T1-T0	*p* H&Y ≥ 3 vs H&Y < 3
D O'CT (s)	−69.5 (−76.0,−62.9)^‡^	−69.0 (−76.7,−61.3)^‡^	−70.1 (−81.3,−58.8)^‡^	0.88
D MMDT placing test 1	−9.6 (−10.8,−8.3)^‡^	−9.0 (−10.4,−7.6)^‡^	−10.2 (−12.4,−8.0)^‡^	0.34
D MMDT placing test 2	−8.4 (−9.8,−7.0)^‡^	−8.6 (−10.2,−7.0)^‡^	−8.3 (−10.7,−5.8)^‡^	0.83
D MMDT placing test total score	−18.5 (−21.0,−16.0)^‡^	−17.2 (−20.0,−14.4)^‡^	−20.0 (−24.3,−15.6)^‡^	0.28
D MMDT turning test 1	−3.9 (−4.4,−3.4)^‡^	−3.7 (−4.2,−3.1)^‡^	−4.2 (−5.2,−3.2)^‡^	0.30
D MMDT turning test 2	−4.2 (−4.8,−3.6)^‡^	−3.7 (−4.3,−3.1)^‡^	−4.8 (−5.9,−3.7)^‡^	0.08
D MMDT turning test 3	−3.0 (−3.5,−2.6)^‡^	−2.9 (−3.3,−2.4)^‡^	−3.3 (−4.0,−2.5)^‡^	0.38
D MMDT turning test 4	−3.2 (−3.7,−2.7)^‡^	−2.9 (−3.4,−2.3)^‡^	−3.6 (−4.4,−2.8)^‡^	0.17
D MMDT turning test total score	−14.1 (−15.7,−12.5)^‡^	−12.8 (−14.5,−11.0)^‡^	−15.7 (−18.6,−12.8)^‡^	0.08
D UPDRS part II	−4.4 (−4.6,−4.2)^‡^	−3.9 (−4.2,−3.7)^‡^	−4.9 (−5.2,−4.6)^‡^	<0.0001
D UPDRS part III	−6.0 (−6.3,−5.8)^‡^	−5.8 (−6.1,−5.5)^‡^	−6.2 (−6.6,−5.9)^‡^	0.09
D SPDDS	−15.8 (−16.6,−15.1)^‡^	−16.0 (−17.0,−15.1)^‡^	−15.6 (−16.8,−14.5)^‡^	0.62

O'CT (O'Connor finger dexterity test); MMDT (Minnesota manual dexterity test); UPDRS (unified Parkinson's disease rating scale); SPDDS (self-assessment Parkinson's disease disability scale); *D* (delta). Reported *p* values are from the Mann-Whitney *U* test (between-group comparison, H&Y ≥ 3 vs H&Y < 3). ^‡^*p* < 0.001, Wilcoxon signed-rank test testing H0: delta = 0 (within-subject). Data are reported as mean (95% CI). For all considered scores, a negative change (i.e., a reduction after MIRT) is associated with an improvement.

**Table 4:** Differences (values at discharge—values at admission) in MMDT and O'CT for patients grouped on the basis of the side of motor symptoms predominance.

Variable	All patients T0	Side left T0	Side right T0	All patients T1	Side left T1	Side right T1	*p* T0	*p* T1
O'CT	499.6 (487.4, 511.8)	496.0 (477.0, 515.0)	498.3 (482.2, 514.4)	436.1 (424.7, 447.5)	434.7 (416.0, 453.3)	433.07 (418.6, 447.4)	0.82	0.62
MMDT placing test 1	103.5 (101.4, 105.6)	101.2 (98.0, 104.4)	105.1 (102.1, 108.1)	94.0 (92.2, 95.9)	92.1 (89.6, 94.6)	95.2 (92.4, 98.0)	0.08	0.41
MMDT placing test 2	97.8 (95.7, 99.9)	95.4 (92.3, 98.5)	99.7 (96.7, 102.7)	89.4 (87.7, 91.1)	87.4 (84.9, 90.0)	90.6 (88.1, 93.0)	0.042	0.14
MMDT placing test total score	201.2 (197.0, 205.3)	196.2 (189.9, 202.5)	204.8 (199.0, 210.7)	182.9 (179.4, 186.3)	178.1 (173.3, 182.8)	185.8 (180.6, 191.0)	0.036	0.12
MMDT turning test 1	24.2 (23.4, 24.9)	24.3 (23.0, 25.6)	23.8 (22.8, 24.7)	20.3 (19.8, 20.9)	20.4 (19.5, 21.2)	20.1 (19.4, 20.9)	0.89	0.76
MMDT turning test 2	25.6 (24.8, 26.5)	25.8 (24.7, 27.0)	25.1 (23.9, 26.2)	21.7 (21.0, 22.4)	21.7 (20.8, 22.7)	21.5 (20.4, 22.6)	0.09	0.31
MMDT turning test 3	21.8 (21.2, 22.5)	21.4 (20.4, 22.3)	21.9 (21.1, 22.8)	18.9 (18.4, 19.4)	18.8 (18.0, 19.5)	18.7 (18.0, 19.3)	0.34	0.80
MMDT turning test 4	22.9 (22.1, 23.6)	23.0 (21.9, 24.2)	22.6 (21.6, 23.5)	19.7 (19.2, 20.3)	19.9 (19.1, 20.8)	19.5 (18.8, 20.2)	0.47	0.60
MMDT turning test total score	94.2 (91.4, 96.9)	94.2 (90.0, 98.5)	93.3 (89.6, 96.9)	80.6 (78.4, 82.7)	80.6 (77.5, 83.7)	79.9 (76.9, 82.9)	0.58	0.67

O'CT (O'Connor finger dexterity test); MMDT (Minnesota manual dexterity test); reported *p* values are from the Mann–Whitney *U* test (*p* T0 and *p* T1, between-group comparison, left-side vs right-side motor symptoms patients at T0 and at T1, respectively). Data are reported as mean (95% CI).

## Data Availability

The Excel file containing the data used to support the findings of this study is available from the corresponding author upon request.
